# Antioxidative Polyphenols from Defatted Oilseed Cakes: Effect of Solvents

**DOI:** 10.3390/antiox3010067

**Published:** 2014-02-24

**Authors:** Sue-Siang Teh, Alaa El-Din Bekhit, John Birch

**Affiliations:** Department of Food Science, University of Otago, PO Box 56, Dunedin 9054, New Zealand; E-Mails: aladin.bekhit@otago.ac.nz (A.E.-D.B.); john.birch@otago.ac.nz (J.B.)

**Keywords:** defatted seed cakes, total phenolics, flavonoids, solvent extraction, antioxidant capacity

## Abstract

Defatted hemp, flax and canola seed cakes were extracted with different solvent systems namely methanol, ethanol, acetone, methanol 80%, acetone 80% and mixed solvent of methanol:acetone:water (MAW, 7:7:6, v/v/v). Each extract was analyzed for antioxidant capacity using ferric reducing/antioxidant power (FRAP) and 2,2-diphenyl-1-picrylhydrazyl (DPPH) radical scavenging assays. MAW exhibited the highest extraction of phenolic and flavonoid contents in the seed cakes, followed by acetone 80% and methanol 80%. The antioxidant capacity was proportional to the polyphenols recovery in the extracts. Canola seed cakes possessed the highest recovery of polyphenols and antioxidant capacity, followed by hemp and flax seed cakes. MAW extract of canola contained total phenolic content, 2104.67 ± 2.52 mg GAE/100 g fresh weight; total flavonoids, 37.79 ± 0.04 mg LUE/100 g fresh weight; percentage inhibition of DPPH^•^, 33.03 ± 0.38%; FRAP assay, 8.78 ± 0.07 μmol Fe (II)/g fresh weight. Identification of individual polyphenol compounds were performed HPLC. MAW extract of canola had the highest (*P* < 0.05) concentration of all individual polyphenols except gallic acid and catechin. Highest concentration of quercetin and luteolin in MAW extract of hemp was obtained among all solvent systems.

## 1. Introduction

Antioxidants are compounds that can postpone oxidation processes or inhibit the propagation stage of free radical reactions in order to protect the body cells from oxidation. They possess abilities such as scavenging free radicals, reducing activity, chelating pro-oxidant metals, inhibiting lipid peroxidation and quenching singlet oxygen [1]. Free radical formation happens constantly in the cells as a result of both enzymatic and non-enzymatic reactions. Enzymatic reactions that distribute free radicals occur in the respiratory chain, phagocytosis, xanthine oxidase, reactions involving iron and other transition metals, arachidonate pathways, peroxisomes, exercise, inflammation, ischaemia/reperfusion, prostaglandin synthesis and cytochrome P450 system [2]. Free radicals are formed in non-enzymatic reactions of oxygen with organic compounds as well as those initiated by ionizing radiations. Free radicals are also generated by externally generated sources such as cigarette smoke, environmental pollutants, radiation, ultraviolet light, certain drugs, pesticides, ozone, anaesthetics and industrial solvents [3]. The damage of all cellular macromolecules including proteins, carbohydrates, lipids and nucleic acids by free radicals lead to degenerative diseases such as cancer, cardiovascular disease, cataracts, Alzheimer’s disease, atherosclerosis, hypertension, diabetes mellitus and aging [4].

Natural antioxidants are available in various forms such as phenolics, flavonoids, coumarins, xanthons, lignans, tannins, curcumanoids, tocopherol, lycopene and β-carotene [5]. They are found in various parts of plants such as fruits, leaves, seeds and oils [6]. The study of antioxidant compounds has drawn the interest of researchers because they are effective in inhibiting free radicals and thus decelerating the formation of degenerative disease. Previous studies show that the antioxidant compounds improve human health such as inhibition of cancer cells, improving the condition of cardiovascular diseases and diabetes [7], healing human chronic ulceration [8], anti-allergenic, anti-artherogenic, anti-inflammatory, anti-microbial, antioxidant, anti-thrombotic, cardioprotective and vasodilatory effects [9].

In this study, antioxidant compounds, namely phenolics and flavonoids in the defatted seed cakes, were studied. Seed cakes are the by-product of cold-pressed oil and are usually used as animal feeds. It is of interest to study the bioactive compounds in the seed cakes which are beneficial to human health. Phenolic compounds are the dominant group of phytochemicals or secondary metabolites that are derived from the pentose phosphate, shikimate, and phenylpropanoid pathways in plants [10]. Flavonoids are a large group of plant phenolics that consist of two aromatic rings that are joined by a 3-carbon bridge in the form of a heterocyclic ring [11].

Previous studies have shown the potential recovery of polyphenols in agrowastes, such as rice hulls [12], buckwheat hulls [13], pistachio hulls [14] and citrus peels, where polyphenol recovery was higher in the peels compared to the edible portion [15]. For seed cakes, most polyphenols were not successfully extracted into the oil after cold-pressing [7].

Several solvent systems have been used to extract polyphenols from plant materials. For example, mixed solvent of methanol:acetone:water (7:7:6, v/v/v) has been used to extract phenolic compounds from de-oiled mesocarp of palm fruit [16]. Methanol, ethanol, acetone, hexane, diethyl ether and petroleum ether have been used to extract phenolic compounds in potato peel, sugar beet pulp and sesame cake [5]. In addition, methanol 80% has been used to extract phenolic compounds in flax seed cakes [7].

The objectives of this study were the determination of the phenolic and flavonoid contents in hemp, flax and canola seed cakes, efficiency of different solvent systems for the extraction of polyphenols in the seed cakes and antioxidant capacity of the polyphenol extracts using the different solvent systems.

## 2. Experimental Section

### 2.1. Chemicals and reagents

Methanol, Acetone and sodium acetate were purchased from Labserv™, Biolab (Aust) Ltd., Victoria, Australia. Hexane and ethanol were purchased from Unilab, Ajax Finechem Pty. Ltd., New South Wales, Australia. Folin-Ciocalteau’s phenol reagent was purchased from Merck, Darmstadt, Germany. Gallic acid, diphenylboric acid 2-aminoethyl ester, ferrous sulphate, 2,4,6-tri(2-pyridyl)-s-triazine (TPTZ) and 1,1-diphenyl-2-picrylhydrazyl (DPPH^•^) were purchased from Sigma Chemical Co., St. Louis, MO, USA. Glacial acetic acid was purchased from ECP Ltd., Auckland, New Zealand. Hydrochloric acid and ferric chloride were purchased from BDH Chemicals Ltd., Poole, England. All chemicals were analytical grade.

### 2.2. Samples

Defatted hemp (*Cannabis sativa*) and flax seed (*Linum usitatissimum*) cakes were donated by Oil Seed Extractions Limited, Ashburton, New Zealand while canola (*Brassica napus*) seed cake was supplied by New Zealand Vegetable Oil Limited, Canterbury, New Zealand. The seed cakes were milled into powder using a Cemotec Sample Mill 1090 (FOSS Tecator, Hoganas, Sweden) and the powders were sieved to produce particles to pass a 450 μm sieve.

### 2.3. Extraction of Polyphenols

Seed cake powders (6 g) were mixed with 100 mL of solvent in a conical flask. The mixtures were stirred with a magnetic stirrer (4.5 × 0.5 cm) at 1000 rpm without application of heat for 1 h at room temperature (25 ± 1 °C). The extracts were filtered through filter paper (0.45 μm; Whatman™) by vacuum. Filtrates were collected into a dark glass bottle and stored at 4 °C prior to analysis. The solvents used in the extraction were methanol; ethanol; acetone; methanol 80%; acetone 80% and methanol:acetone:water (MAW; 7:7:6; v/v/v) according to the previous reports [5,16,17,18].

### 2.4. Total Phenolic Content

The determination of total phenolic content in the extracts was based on the method of Gutfinger (1981) [19]. Firstly, 0.1 mL of extract was made up to 5 mL with distilled water in a 10-mL volumetric flask, followed by addition of 0.5-mL 2 N Folin-Ciocalteau’s phenol reagent. About 1 mL of saturated (35% w/v) sodium carbonate solution was added into the mixture after three minutes. The mixture was made up to 10 mL with water. After 1 hour, the mixture was measured spectrophotometrically at 725 nm against the reagent blank. Gallic acid within the concentration range of 0–400 μg/mL assay solution was used as the standard curve for the total phenolic acids content. Results were expressed as mg gallic acid equivalents (GAE)/100 g of fresh weight.

### 2.5. Total Flavonoids

The determination of flavonoids was based on the method of Oomah, Mazza, and Kenaschuk [20]. Extract (1 mL) was mixed with 3 mL of distilled water. Then, 100 μl of diphenylboric acid 2-aminoethyl ester solution (1% v/v) was added into the extract before it was measured spectrophotometrically at 404 nm. Luteolin within the concentration range of 0–42 μg/3 mL assay solution in 80% methanol was used as the standard for the calibration curve. Results were expressed as mg luteolin equivalents (LUE)/100 g of fresh weight.

### 2.6. DPPH Free Radical-Scavenging Assay

The determination of the DPPH free radical-scavenging assay was based on the method of de Ancos *et al.* [21] with some modification. Extract (10 μL) was mixed with 3.99 mL of 25 mM DPPH^•^ methanolic solution. The mixture was vortexed and kept in the dark for 30 min before measurement spectophotometrically at 515 nm, against a blank of methanol without DPPH^•^. Results were expressed as percentage inhibition of the DPPH^•^ using the following equation:

% inhibition of DPPH^•^ = 100 × [(Absorbance control − Absorbance sample)/Absorbance control] (1)

where absorbance control is the absorbance of DPPH^•^ solution without extract.

### 2.7. Ferric Reducing/Antioxidant Power (FRAP) Assay

FRAP assay was based on the method of Benzie and Strain [22] with modification. FRAP reagent was prepared by mixing 200 mL of 300 mM, pH 3.6 acetate buffer, 20 mL of 10 mM 2,4,6-tri(2-pyridyl)-s-triazine (TPTZ) solution, 20 mL of 20 mM FeCl_3_ solution and 24 mL distilled water. TPTZ and FeCl_3_ solutions were made freshly every day prior to analysis. The FRAP reagent was straw in colour and kept in a water bath at 37 °C prior to analysis. Briefly, 40 μL of extract was mixed with 3 mL of FRAP reagent and the reaction mixture was incubated at 37 °C for 4 min before it was measured spectrophotometrically at 593 nm against the blank. The blank solution consisted of 40 μL distilled water in 3 mL FRAP reagent, incubated at 37 °C for 1 h. Standard solutions consisted of FeSO_4_·7H_2_O in different concentrations (0.1, 0.2, 0.4, 0.6, 0.8 and 1.0 mM). The results were expressed as μmol Fe (II)/g fresh weight.

### 2.8. Identification of Polyphenols by HPLC

Separation and identification of phenolic acids were carried out by high-performance liquid chromatography (Varian 9010, California, CA, USA) based on the previous method [23]. The HPLC was equipped with diode array detection system, chromatography software and a NovaPak C18 reversed phase (3.9 × 150 mm, 5 μm) (Waters, Milford, MA, USA) that was set at 35 °C. Solution A was 50 mM sodium phosphate buffer (pH 3.3) and 10% methanol while solution B was 70% methanol. The flow rate was 1 mL/min. The gradient profile was 100% of solution A (15 min), 70% solution A (15 min), 65% solution A (30 min), 60% solution A (20 min), 50% solution A (5 min), and finally 0% solution A (25 min). The chromatograms were recorded at 250, 280 and 320 nm. The identification was carried out by retention time, and the concentration of phenolic acids was determined using external and internal standards of the individual phenolic acids. All solutions were HPLC grade and were filtered prior to HPLC analysis. The extracts (5 mL) was evaporated and dissolved with 1 mL solution B. Injection samples were 10 μL.

### 2.9. Statistical Analysis

Results were reported as mean ± standard deviation of triplicate measurements. Significant difference (*P* < 0.05) within means was analyzed by analysis of variance (ANOVA) and Tukey’s honestly significant difference (HSD) test in the SPSS Statistics Software Version 20 (IBM, New York, NY, USA).

## 3. Results and Discussion

### 3.1. Effect of Solvent System

Total phenolics (TPC ) and total flavonoids (TFC) are the standard method of assessing antioxidant capacity and represents a wide range of individual compounds from phenolic acids to polymers [1,4,5]. Results show solvents were significantly different (*p* < 0.05) in extracting phenolic compounds in the seed cakes (Table 1). The solvent mixture of methanol:acetone:water (MAW) was the best solvent system in extracting the highest yield of phenolic compounds in the seed cakes amongst all solvents. Results showed that total phenolic contents of hemp, flax and canola seed cakes extracted by MAW were 733.33 ± 1.53, 774.33 ± 2.08 and 2104.67 ± 2.52 mg GAE/100 g fresh weight, respectively. The total phenolics of seed cakes in this study was higher than the total phenolics in cold-pressed oils of hemp (2.45 ± 0.05 mg of caffeice acid equivalent (CAE)/100 g sample), flax (1.14 ± 0.03 mg CAE/100 g sample) and canola (1.31 ± 0.04 mg CAE/100 g sample) based on a previous report [24]. The second best solvent was acetone 80%, followed by methanol 80%, acetone, methanol, ethanol and hexane. Each solvent possesses various degrees of polarity that resulted in different extraction strengths. In addition, it was found that solvents in aqueous environments such as methanol 80% and acetone 80% had stronger extraction strength compared to the solvent alone. This was due to the increase in polarity of the solvent system in the presence of water. Acetone 50% and ethanol 70% yielded the highest total phenolic content in pineapple while acetone 70% resulted in the highest yield of total phenolic content in banana [17]. Acetone-water mixtures also proved to be efficient solvent systems for extracting polar antioxidants according to [25,26]. Hexane was the weakest solvent in extracting phenolic compounds from the seed cakes (Table 1). This is supported by a previous study that showed that hexane yielded the lowest total phenolic content and antioxidant capacity in seed cakes, from evening primrose, burdock, sesame, and woad [18]. They found that hexane is only suitable to extract phosphatides, lipid and other fat soluble components such as tocopherols, tocotrienols and carotenoids but it is too weak to extract hydrophilic phenolic compounds due to low polarity of hexane. Whereas hexane resulted in the highest oil yield from flaxseed cake compared to other solvents [27], the authors found that a mixed solvent of methanol:ammonia:hexane (90:5:5 v/v/v) resulted in lower oil yield. Hence, there is a contrast for the types and polarity of solvent for extracting a specific component in the seed cakes. In order to increase the extraction of total phenolic content in the seed cakes, the polarity of hexane needs to be increased by mixing with other polar solvents such as methanol, acetone and ethanol.

**Table 1:** Total phenolic content (mg GAE/100 g fresh weight).

Solvent	Seed Cakes		
	Hemp	Flax	Canola
Methanol	432.33 ± 2.52 ^eB^	275.00 ± 1.00 ^eC^	1776.33 ± 1.53 ^cA^
Ethanol	351.33 ± 2.08 ^fB^	255.00 ± 3.00 ^fC^	1025.00 ± 2.65 ^eA^
Hexane	167.00 ± 2.00 ^gB^	108.33 ± 0.58 ^gC^	488.67 ± 7.02 ^fA^
Acetone	483.67 ± 3.51 ^dB^	374.67 ± 3.51 ^dC^	1390.67 ± 4.04 ^dA^
Acetone 80%	642.67 ± 0.58 ^bB^	564.67 ± 2.52 ^bC^	1976.33 ± 1.53 ^bA^
Methanol 80%	545.33 ± 2.08 ^cB^	406.67 ± 2.08 ^cC^	1987.33 ± 2.08 ^bA^
Methanol:acetone:water (7:7:6 v/v/v)	733.33 ± 1.53 ^aC^	774.33 ± 2.08 ^aB^	2104.67 ± 2.52 ^aA^

Results are expressed as mean ± standard deviations; *n* = 3. ^a–g^ Means in the same column followed by different lowercase letters are significantly different (*P* < 0.05). ^A–C^ Means in the same row followed by different uppercase letters are significantly different (*P* < 0.05).

The flavonoid content in the seed cakes extracted by different solvents was significantly different (*p* < 0.05) and followed the same order of solvents used in extracting phenolic compounds (Table 2), namely MAW > acetone 80% > methanol 80% > acetone > methanol > ethanol > hexane. The mixed solvent, MAW yielded 27.41 ± 0.04, 9.18 ± 0.17, 37.79 ± 0.04 mg LUE/100 g fresh weight of hemp, flax and canola seed cakes, respectively.

**Table 2:** Total flavonoids (mg LUE/100 g fresh weight).

Solvent	Seed Cakes		
	Hemp	Flax	Canola
Methanol	3.86 ± 0.06 ^eB^	3.47 ± 0.06 ^eC^	28.13 ± 0.06 ^dA^
Ethanol	0.81 ± 0.06 ^fB^	0.58 ± 0.08 ^fB^	25.28 ± 0.42 ^eA^
Hexane	0.23 ± 0.10 ^gB^	0.08 ± 0.05 ^gB^	20.27 ± 0.57 ^fA^
Acetone	4.31 ± 0.06 ^dB^	4.33 ± 0.09 ^dB^	28.63 ± 0.09 ^dA^
Acetone 80%	8.04 ± 0.07 ^bB^	5.36 ± 0.11 ^bC^	36.05 ± 0.12 ^bA^
Methanol 80%	7.61 ± 0.04 ^cB^	5.01 ± 0.18 ^cC^	35.03 ± 0.05 ^cA^
Methanol:acetone:water (7:7:6 v/v/v)	27.41 ± 0.04 ^aB^	9.18 ± 0.17 ^aC^	37.79 ± 0.04 ^aA^

Results are expressed as mean ± standard deviations; *n* = 3. ^a–g^ Means in the same column followed by different lowercase letters are significantly different (*P* < 0.05). ^A–C^ Means in the same row followed by different uppercase letters are significantly different (*P* < 0.05).

### 3.2. Antioxidant Capacity

There are many assays to evaluate antioxidant capacity of plant materials. For example, DPPH free radical-scavenging assay, ferric reducing/antioxidant power (FRAP) assay and β-carotene bleaching model [28]. Each assay has its own specific mechanism to measure the antioxidant capacity of plant materials, where the concept of the antioxidant capacity evaluation is based on the ability of the antioxidant to scavenge free radical compounds since free radicals are a main cause of the propagation phase in the oxidation process. In this study, DPPH^•^ free radical-scavenging and FRAP assays were used to evaluate the antioxidant extracts in the seed cakes.

DPPH^•^ is a stable organic nitrogen radical (1,1-diphenyl-2-picrylhydrazyl). In order to evaluate the antioxidant capacity of the extracts from various seed cakes, DPPH^•^ free radicals are reduced by an antioxidant extract to their hydroxyl group within the assay time, thus the remaining DPPH^•^ free radicals are measured spectrophotometrically at 515 nm. A previous study reported that DPPH^•^ free radical scavenging assay represents the ability of the antioxidant extract to scavenge free radicals through hydrogen- or electron-donating mechanisms [5].

FRAP assay is another conventional method used in evaluating the antioxidant capacity of plant extracts. The principle of the FRAP assay is based on the antioxidant strength in reducing ferric-tripyridyltriazine complex to its ferrous form. The intensity of the blue colour formation is proportional to the concentration of the ferrous form and the antioxidant capacity of the extract. Antioxidant compounds that exhibit antioxidant capacity in FRAP assay are usually electron donors as they reduce the oxidized intermediates to the stable form in order to eliminate the oxidation chain reaction [1].

#### 3.2.1. DPPH Free Radical-Scavenging Assay

Previous study reported that change in solvent polarity by mixing different types of solvents transforms its capability to extract specific groups of antioxidant compounds that can affect the antioxidant capacity since antioxidant compounds of various polarities could exist in the seed cakes [29]. Significant differences (*p* < 0.05) of % inhibition DPPH^•^ between extracts using the different solvents were found in this study (Table 3). The results showed that MAW extracts exhibited the highest % inhibition DPPH^•^, reported as 16.79 ± 0.09, 11.39 ± 0.09 and 33.03 ± 0.38% inhibition DPPH^•^ for hemp, flax and canola seed cakes extracts, respectively. Extracts with acetone 80% and methanol 80% exhibited the second and third highest % inhibition DPPH^•^, respectively. The results indicated that the antioxidant capacity of the seed cake extracts with aqueous acetone and aqueous methanol were superior compared to the seed cake extracts with pure solvents. Extracts with hexane possessed lowest antioxidant capacity. This was supported by previous studies that showed that extracts of potato peel, sugar beet pulp, sesame cake [5] and medicinal plants [30] extracted with hexane were far less effective in scavenging free radicals. Hence, this showed that high-polarity solvents were more effective to extract antioxidant compounds that exhibited more efficient radical scavenging property compared to low-polarity solvents.

Previous report showed that about 0.5 mg of canola or hemp seed cake extract scavenged the DPPH radicals by 50% [31], which indicates that MAW extract of canola and hemp seed cake exhibited higher DPPH^•^ scavenging activity compared to flax seed cake extract. Although the previous report by Matthäus (2002) [31] showed higher DPPH^•^ scavenging activity than the study here, it should be noted that the different extraction method and sample amount affected the antioxidant capacity of the extract, as the author performed three extractions for 15 g of seed cake with 150 mL of solvent that consisted of 16 h shaking and two times of 45 min each ultrasonic extraction. In this study, simple shaking extraction for 1 h using different solvent systems was performed with respect to the aims of the study here.

**Table 3:** DPPH free radical-scavenging assay (% inhibition of DPPH^•^).

Solvent	Seed Cakes		
	Hemp	Flax	Canola
Methanol	5.33 ± 0.30 ^eB^	4.42 ± 0.26 ^cC^	26.60 ± 0.22 ^dA^
Ethanol	3.09 ± 0.23 ^fB^	3.58 ± 0.22 ^dB^	15.07 ± 0.46 ^eA^
Hexane	1.93 ± 0.32 ^gB^	1.72 ± 0.47 ^eB^	6.59 ± 0.26 ^fA^
Acetone	8.68 ± 0.10 ^dB^	3.09 ± 0.15 ^dC^	28.04 ± 0.17 ^cA^
Acetone 80%	12.48 ± 0.46 ^bB^	7.81 ± 0.10 ^bC^	29.29 ± 0.38 ^bA^
Methanol 80%	11.01 ± 0.23 ^cB^	7.36 ± 0.10 ^bC^	28.54 ± 0.17 ^bcA^
Methanol:acetone:water (7:7:6 v/v/v)	16.79 ± 0.09 ^aB^	11.39 ± 0.09 ^aC^	33.03 ± 0.38 ^aA^

Results are expressed as mean ± standard deviations; *n* = 3. ^a–g^ Means in the same column followed by different lowercase letters are significantly different (*P* < 0.05). ^A–C^ Means in the same row followed by different uppercase letters are significantly different (*P* < 0.05).

#### 3.2.2. Ferric Reducing/Antioxidant Power (FRAP) Assay

FRAP assay of the seed cakes extracts had a similar trend to the DPPH^•^ assay comparing the solvents used in extraction of the seed cakes. Significant differences (*p* < 0.05) of ferric reducing value between extracts extracted with different solvents are shown in Table 4. The results show that extracts with MAW exhibited the highest reducing power, 3.51 ± 0.04, 1.48 ± 0.00, 8.78 ± 0.07 μmol Fe (II)/g fresh weight for hemp, flax and canola seed cakes, respectively. Again, extracts with hexane exhibited the lowest reducing power with the aqueous polar solvents proving superior to the pure polar solvents.

Overall, results show that antioxidant capacity was in accordance with the contents of phenolic and flavonoid compounds in the seed cake extracts, which was similar to previous findings [32,33]. It appears that the flavonoids have had a greater influence on the antioxidant capacity for hemp seed cake compared to flax seed cake where the phenolic content was marginally higher with the MAW solvent system.

**Table 4:** Ferric reducing/antioxidant power (FRAP) assay (μmol Fe (II)/g fresh weight).

Solvent	Seed Cakes		
	Hemp	Flax	Canola
Methanol	1.11 ± 0.02 ^eB^	0.60 ± 0.01 ^eC^	3.46 ± 0.03 ^cdA^
Ethanol	1.03 ± 0.05 ^eB^	0.51 ± 0.01 ^fC^	2.45 ± 0.04 ^dA^
Hexane	0.07 ± 0.02 ^fB^	0.03 ± 0.00 ^gC^	1.20 ± 0.02 ^dA^
Acetone	2.09 ± 0.05 ^dB^	0.64 ± 0.01 ^dC^	5.15 ± 2.36 ^bcA^
Acetone 80%	3.19 ± 0.03 ^bB^	1.10 ± 0.01 ^bC^	8.15 ± 0.06 ^aA^
Methanol 80%	2.41 ± 0.08 ^cAB^	0.93 ± 0.02 ^cC^	7.18± 0.18 ^abA^
Methanol:acetone:water (7:7:6 v/v/v)	3.51 ± 0.04 ^aB^	1.48 ± 0.00 ^aC^	8.78 ± 0.07 ^aA^

Results are expressed as mean ± standard deviations; *n* = 3. ^a–g^ Means in the same column followed by different lowercase letters are significantly different (*P* < 0.05). ^A–C^ Means in the same row followed by different uppercase letters are significantly different (*P* < 0.05).

**Figure 1:**
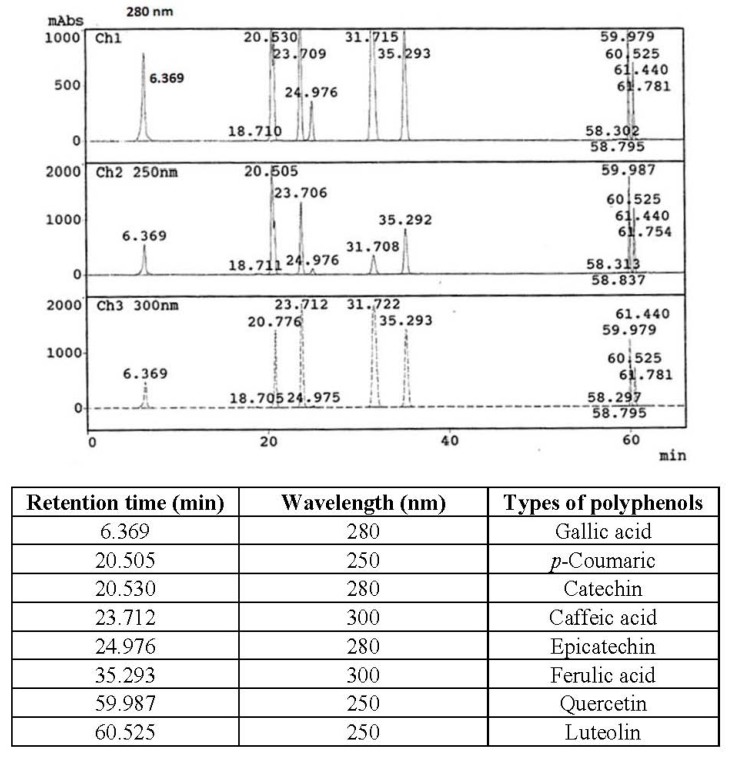
Polyphenols mixed standard of HPLC.

The identification of polyphenol compounds was based on the polyphenols mix standard that shown in Figure 1. Polyphenol compounds of defatted hemp, flax and canola seed cakes are shown in Table 5. There were other unidentified polyphenol compounds found in the extracts by HPLC in addition to the compounds shown in Figure 1. Hence, the total of individual polyphenol compounds did not represent the total phenolics and total flavonoids in the extract.

Previous studies reported the main polyphenols in canola seed oil and cake were sinapic acid and tannin [34,35], in which they are the main components that exert antioxidant properties. However, there was little information on polyphenols composition of defatted canola seed cake. In this study, more individual polyphenol compounds were identified in defatted canola seed cake extract in all seed cake extracts. Epicatechin appeared to be the highest (*P* < 0.05) compound in defatted canola seed cake extract, followed by caffeic acid. Gallic acid appeared moderately in defatted canola seed cake extract, ranging 76.19–88.98 mg/100 g fresh weight, followed by catechin and luteolin. The least (*P* < 0.05) compound was *p*-coumaric, ranging 24.04–34.22 mg/100 g fresh weight.

MAW was the most efficient solvent system for the highest yield of polyphenols as MAW was made of different polarities of solvents. Hence, MAW was able to extract polyphenols of different polarity. Other studies reported that the presence of water in solvent increased polyphenols extraction efficiency compared to pure solvent [35,36], which was similar to the results here. As shown in Table 5, the low polarity of hexane was not able to extract high polarity polyphenol compounds such as gallic acid, *p*-coumaric and ferulic acid in defatted canola seed cake extract. Methanol 80% extracted the highest concentration (*P* < 0.05) of gallic acid and catechin in defatted canola seed cake due to high polarity of these polyphenol compounds. However, the high polarity of methanol 80% was not efficient in extracting less polar polyphenol compounds such as quercetin, luteolin and ferulic acid.

Lignan is a major component of polyphenol in defatted flax seed cake extract [37]. Other detected phenolic acids of defatted flax seed cake such as *p*-coumaric, cinnamic acid, ferulic acid and caffeic acid have been reported previously [38,39,40]. In this study, *p*-coumaric and ferulic were detected by HPLC. Due to high polarity of *p*-coumaric and ferulic acid in defatted flax seed cake, the highest polarity of methanol 80% was the most efficient solvent system in this case. However, it should be noted that this was not reflected in total phenolics and total flavonoids in the extracts.

Previous studies reported the antioxidant properties and total phenolics of hemp seed oil [41]. Although there was limited information of polyphenol compounds in defatted hemp seed cake extract, three types of compounds—caffeic acid, quercetin and luteolin in defatted hemp seed cake, were detected in this study. Quercetin appeared to be the highest (*P* < 0.05) compound, followed by luteolin, while caffeic acid had the lowest (*P* < 0.05) concentration in defatted hemp seed cake extract (Table 5). Methanol and methanol 80% extracted the highest concentration of caffeic acid in defatted hemp seed cake due to the high polarity of the solvent system and phenolic compound. Nevertheless, MAW appeared to be the most efficient (*P* < 0.05) solvent system in extracting quercetin and luteolin which are the major polyphenols in the defatted hemp seed cake extract.

**Table 5:** Identification polyphenols of defatted oilseed cakes by HPLC.

	Polyphenol compounds (mg/100 g fresh weight)							
	Gallic acid	*p*-Coumaric	Catechin	Caffeic acid	Epicatechin	Ferulic acid	Quercetin	Luteolin
*Defatted hemp seed cake extracts*								
A	-	-	-	8.31 ± 0.02 ^aC^	-	-	62.11 ± 0.19 ^fA^	40.15 ± 0.12 ^dB^
B	-	-	-	7.90 ± 0.10 ^bC^	-	-	66.02 ± 0.30 ^dA^	37.69 ± 0.34 ^eB^
C	-	-	-	-	-	-	44.69 ± 0.34 ^gA^	44.15 ± 0.17 ^bB^
D	-	-	-	6.00 ± 0.20 ^dC^	-	-	80.00 ± 0.15 ^bA^	42.15 ± 0.17 ^cB^
E	-	-	-	7.28 ± 0.07 ^cC^	-	-	74.19 ± 0.17 ^cA^	44.11 ± 0.19 ^bB^
F	-	-	-	8.27 ± 0.04 ^aC^	-	-	64.89 ± 0.14 ^eA^	40.04 ± 0.08 ^dB^
G	-	-	-	5.37 ± 0.12 ^e^	-	-	104.11 ± 0.19 ^aA^	46.11 ± 0.19 ^aB^
*Defatted flax seed cake extract*								
A	-	10.15 ± 0.17 ^bA^	-	-	-	5.60 ± 0.25 ^bB^	-	^-^
B	-	10.05 ± 0.07 ^bA^	-	-	-	5.05 ± 0.12 ^bB^	-	^-^
C	-	-	-	-	-	-	-	^-^
D	-	6.13 ± 0.20 ^eA^	-	-	-	4.11 ± 0.19 ^cB^	-	^-^
E	-	7.11 ± 0.19 ^dA^	-	-	-	5.22 ± 0.19 ^bB^	-	^-^
F	-	12.15 ± 0.17 ^aA^	-	-	-	6.22 ± 0.19 ^aB^	-	^-^
G	-	9.04 ± 0.08 ^cA^	-	-	-	5.56 ± 0.25 ^bB^	-	^-^
*Defatted canola seed cake extract*								
A	88.11 ± 0.19 ^bC^	32.44 ± 0.77 ^bG^	50.19 ± 0.17 ^dE^	447.33 ± 0.58 ^cB^	838.33 ± 0.58 ^cA^	14.11 ± 0.19 ^bH^	63.33 ± 0.58 ^cdD^	38.67 ± 1.16 ^dF^
B	86.15 ± 0.17 ^cC^	28.11 ± 0.19 ^dG^	50.11± 0.19 ^dE^	439.67 ± 0.58 ^dB^	786.22 ± 0.19 ^fA^	14.22 ± 0.19 ^bF^	68.67 ± 1.16 ^bD^	50.11 ± 0.20 ^aE^
C	-	-	9.95 ± 0.11 ^fE^	82.00 ± 0.15 ^fB^	157.22 ± 0.19 ^gA^	-	53.00 ± 1.00 ^eC^	48.11 ± 0.20 ^bD^
D	76.19 ± 0.17 ^dC^	24.04 ± 0.08 ^eG^	46.06 ± 0.24 ^eF^	405.00 ± 2.00 ^eB^	832.44 ± 0.51 ^dA^	9.11 ± 0.19 ^eH^	67.44 ± 0.51 ^bD^	50.44 ± 0.51 ^aF^
E	86.11 ± 0.19 ^cC^	30.15 ± 0.17 ^cG^	52.11 ± 0.19 ^bE^	442.11 ± 0.19 ^dB^	825.22 ± 0.19 ^eA^	12.11 ± 0.19 ^cH^	65.11 ± 0.19 ^cD^	48.11 ± 0.19 ^bF^
F	88.98 ± 0.04 ^aC^	32.11 ± 0.19 ^bG^	52.95 ± 0.20 ^aE^	452.67 ± 2.52 ^bB^	857.11 ± 0.19 ^bA^	11.22 ± 0.19 ^dH^	62.29 ± 0.27 ^dD^	41.44 ± 0.19 ^cF^
G	88.11 ± 0.19 ^bC^	34.22 ± 0.19 ^aF^	51.22 ± 0.19 ^cE^	479.09 ± 0.21 ^aB^	1368.66 ± 1.53 ^aA^	15.55 ± 0.39 ^aG^	72.22 ± 0.19 ^aD^	51.22 ± 0.19 ^aE^

Results are expressed as mean ± standard deviations; *n* = 3. ^a–h^ Means in the same column followed by different lowercase letters are significantly different (*P* < 0.05). ^A–F^ Means in the same row followed by different uppercase letters are significantly different (*P* < 0.05).

## 4. Conclusions

The recovery of phenolic and flavonoid compounds from the seed cakes depended on the type of seed cakes and solvents used in the extraction. Mixed solvent MAW was found to be the most effective solvent system that gave the highest yield of phenolic and flavonoid compounds in seed cakes. Furthermore, the antioxidant capacity in the extract was proportional to the phenolic and flavonoid compounds in the extract. Hence, extracts with MAW possessed the highest reducing power and % inhibition DPPH^•^ since they had the highest phenolic and flavonoid contents.
